# News and Events of the Week

**Published:** 1911-05-20

**Authors:** 


### News and Events of the Week.

The Duke of Connaught has consented to open the newly erected Cancer Research Institute at the Cancer Hospital on Tuesday next, May 23, at 3 o'clock.

At the recent primary examination for the Fellowship in Anatomy and Physiology of the Royal College of Surgeons of England 135 candidates presented themselves, and among those approved was one lady, Miss Eleanor Davies-Colley, M.D., B.S. Lond.

The eighteenth annual report of the London Spectacle Mission Society, which was founded in 1886 by Dr. Waring and which supplies spectacles free of charge to necessitous persons recommended by subscribers, makes mention of the progress done during the past year. The fund needs more support than it gets at present, for it is doing excellent work. The secretary's address is 197 Sutherland Avenue, W.

The Oxford and Kingston steamers begin their daily excursions on May 17. These steamers have now been running more than twenty years, and their popularity has increased with every season. The Great Western Railway, London and South Western Railway, and Messrs. Thos. Cook and Sons book numerous circular tours in connection with this service, thus giving facilities to tourists to take long or short trips over the ninety miles of river served by the steamers, and enabling visits to be made to Hampton Court, Windsor, Maidenhead, Marlow, Henley, Pangbourne, Goring, etc., in the most pleasant of ways.

The Chemists' Exhibition, which the profession and the trade owe to the pioneering effort of the *British and Colonial Druggist*, was this year held at the Holland Park Skating Rink. As in former years, it was an astoundingly complete and interesting exhibition. A walk round the rooms revealed the great strides which have been made during the past twelve months. Not only has there been a vast increase in the number of new preparations, but there is also to be noticed a commendable improvement in quality and in attractiveness. Lack of space unfortunately prevents us from dealing with the exhibition in detail, and indeed it would need more than one article to do justice to its excellence.

The English Committee of the Society of Veterans, who fought in the wars for the unity and independence of Italy, is making an appeal for funds towards the cost of a visit to Rome of the survivors of the British Legion. The veterans are to leave London on June 1 to take part in the Jubilee celebrations. The men are the survivors of the campaign of 1860-61, who landed at Naples from the steamers *Emperor* and *Melazzo*, 674 strong of all ranks. Subscriptions should be sent to the Hon. Treasurer, Baron J. C. Keen, 15 Oxford Street, or the London County and Westminster Bank, Oxford Street Branch.

Messrs. Eyre and Spottiswoode (Bible Warehouse), Ltd., have just published the forms of prayer with thanksgiving to Almighty God, commended by the Archbishops of Canterbury and York for general use on Thursday, June 22, 1911 (Coronation Day), together with the musical service edited by Sir Frederick Bridge. This is the edition of the service recommended for use in all churches on June 22 next, and is being issued in conjunction with Messrs. Novello and Co., Ltd. The price is 1s.

On the initiative of the Metropolitan Counties Branch of the British Medical Association, a complimentary dinner to Mr. Butlin, the President of the Association, has been arranged to take place on June 13 next. The dinner will take place at the Connaught Rooms at 7.30 p.m., and the price of the dinner ticket, exclusive of wine, has been fixed at half a guinea. It is hoped that everyone who desires to be present will make immediate application to the Honorary Secretaries, Butlin Dinner Committee, 429 Strand, W.C.

The growing importance of medical work in connection with Foreign Missions was well shown at the annual meeting of the Medical Mission Auxiliary of the Church Missionary Society held in the Queen's Hall, Langham Place, W. Sir Alfred Pearce Gould, K.C.V.O., M.S., presided over a large and enthusiastic gathering, and the speakers included Messrs. A. E. Druitt, M.R.C.S., L.R.C.P., D.P.H. Camb., of Onitsha, West Africa, A. Neve, F.R.C.S. Edin., of Kashmir, and Nurse Baldwin, of Foochow. The Society had now a staff of eighty-eight medical men and women, and fifty-four fully trained nurses, besides a large staff of native doctors, hospital assistants, and nurses. It maintains fifty hospitals in various parts of the world, and a notable feature of the year's progress has been the consolidation of work by rebuilding, enlarging, and more fully equipping several of these. The work of medical missionaries has been officially recognised by the bestowal of the Kaisar-i-Hind gold medal upon T. L. Pennel, Esq., M.D. Lond., F.R.C.S., of Bannu, and the silver medal of the same Order upon H. T. Holland, Esq., M.B., Ch.B., F.R.C.C. Edin., of Quetta.

Radiography again bulks largely in the scientific section of the Royal Photographic Society's exhibition, which opened at the Prince's Skating Club, Knightsbridge, on May 9th. Dr. C. Thurstan Holland, of Liverpool, obtains a medal for two radiographs, one showing bismuth food in a prolonged transverse colon, and the other the bismuth meal in a stomach with hour-glass constriction. The same worker is responsible for several radiographs showing stones in the kidney, and for one example of the result of injecting collargol solution into a cystic kidney. There are several examples of what may be called snapshot radiography. A radiograph revealing tuberculous disease of the lung, exhibited by Dr. Robert Knox, had an exposure of a hundredth of a second, with the new Dessauer apparatus, and a similar exposure was given in the case of a bismuth meal in the dilated œsophagus. Dr. G. H. Rodman contributes a series of three radiographs which illustrate the advantage of using an intensifying screen in contact with the emulsion of the plate at the time of exposure. Two radiographs by Dr. G. T. Haenisch, of Hamburg, should also be noted. One of these shows how an early diagnosis was made of a cancer in the colon sigmoideum, subsequently proved by operation. A bismuth enema was injected into the rectum, and the plate shows the open space in the S. Romanum, where the tumour prevented the filling by the bismuth. Dr. Haenisch's other radiograph shows a kink in a large S. Romanum which produced a spasmus higher up. The exposures in both these cases were about half a second. The radiograph of the head of a child of twelve years showing the formation of the second teeth is placed on exhibition by Mr. William Jolly and Mr. J. Inderwick Pigg.

